# Addressing the role of the α-helical extension in the folding of the third PDZ domain from PSD-95

**DOI:** 10.1038/s41598-017-12827-0

**Published:** 2017-10-03

**Authors:** Candice Gautier, Lorenzo Visconti, Per Jemth, Stefano Gianni

**Affiliations:** 1 Istituto Pasteur - Fondazione Cenci Bolognetti, Dipartimento di Scienze Biochimiche “A. Rossi Fanelli” and Istituto di Biologia e Patologia Molecolari del CNR, Sapienza Università di Roma, 00185 Rome, Italy; 2 Department of Medical Biochemistry and Microbiology, Uppsala University, BMC Box 582, SE-75123 Uppsala, Sweden

## Abstract

PDZ domains are one of the most important protein-protein interaction domains in human. While presenting a conserved three dimensional structure, a substantial number of PDZ domains display structural extensions suggested to be involved in their folding and binding mechanisms. The C-terminal α-helix extension (α3) of the third PDZ domain from PSD-95 (PDZ3) has been reported to have a role in function of the domain as well as in the stabilization of the native fold. Here we report an evaluation of the effect of the truncation of this additional helix on the folding and unfolding kinetics of PDZ3. Fluorescent variants of full length and truncated PDZ3 were produced and stopped-flow fluorescence measurements were made under different experimental conditions (pH, ionic strength and temperature) to investigate the folding kinetics of the respective variant. The results show that folding of PDZ3 is robust and that the mechanism is only marginally affected by the truncation, which contributes to a destabilization of the native state, but otherwise do not change the overall observed kinetics. Furthermore, the increase in the unfolding rate constants, but not the folding rate constant upon deletion of α3 suggests that the α-helical extension is largely unstructured in the folding transition state.

## Introduction

**Figure 1:**
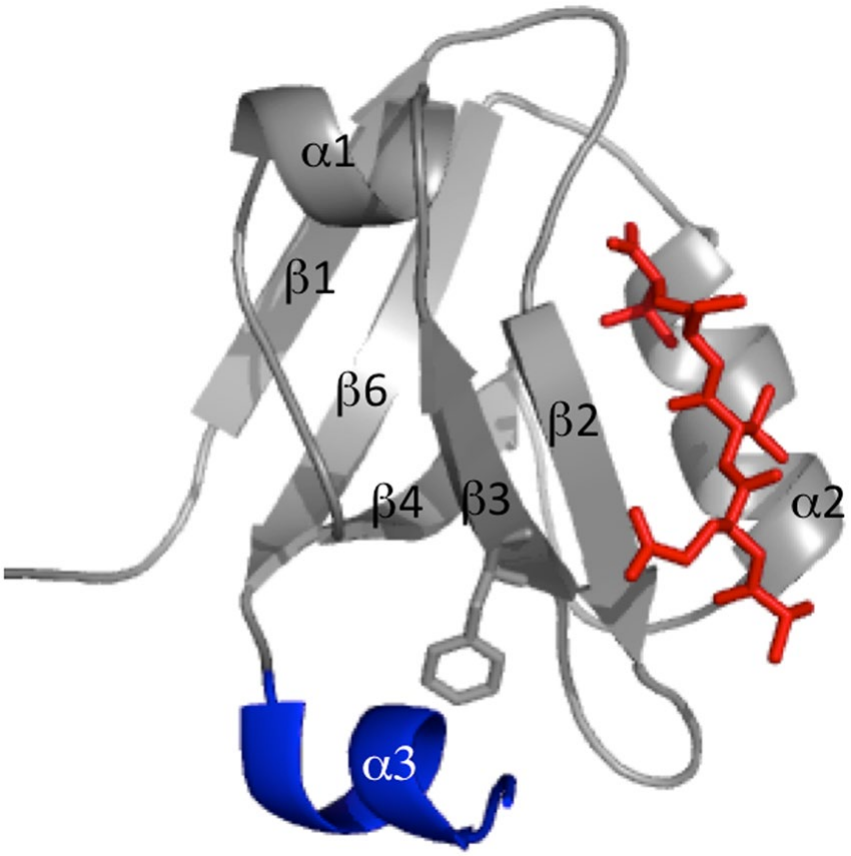
Three dimensional structure of PSD-95 PDZ3 domain (pdb code 1BE9) in complex with a peptide ligand, YKQTSV. The conserved structure of PDZ domains is colored in grey, the additional C-terminal α-helix is highlighted in blue and the backbone of the peptide is colored in red. Residue number 337, which was mutated into Trp as a probe for fluorescence monitored folding studies, is highlighted in sticks.

PDZ domains are the most abundant protein interaction modules in human, being characterized by over 200 different domains^1–5^. Their function is to recognize a specific partner and occurs by binding to short amino acid sequences (PDZ binding motifs), typically located at the carboxyl terminus of the target polypeptide^6,7^. The three-dimensional structure of PDZ domains is highly conserved and is characterized by a globular fold of about 90 residues, composed of six β-strands and two α-helices^8^; the six β-strands form two antiparallel β-sheets stacked onto each other (Fig. 1), with the binding pocket placed between helix α2 and strand β2.

PDZ domains are often present in tandem repeats and several reports suggested that their function is slightly affected when they are studied in isolation rather than in the context of a multi-domain protein^9,10^. Such features occur via the propagation of energetic signals from the binding site to physically distinct regions in the domain. Consequently, the PDZ domain family has been suggested to display some allosteric features, which, while being relatively subtle, would be the basis of their fine regulation^11–14^. These allosteric features have been described invoking both structural and dynamic changes, and have been detected using several experimental and *in silico* methods including statistical coupling analysis^15^, molecular dynamics simulations^16^, NMR^17^ and double mutant cycles used in synergy with binding kinetics^13,18^.

The simplest paradigmatic example of inter-domain regulation of a PDZ domain is represented by the role of the C-terminal extension in the third PDZ domain from PSD-95 (PDZ3), which is an α-helix (α3) (Fig. 1). In fact, a comparison by NMR between the full-length and truncated constructs, where this contiguous structural element was deleted, suggested α3 to be responsible for the modulation of dynamic and binding properties of the PDZ domain^17,19,20^. Such a behavior was mainly assigned to the dynamic properties of the side chain of the domain, and led the authors to propose α3 to represent an element of allosteric regulation for PDZ3. Notably, however, a comprehensive analysis of the role of α3 by protein engineering in conjunction with NMR, ITC, and fluorescence based kinetic experiments revealed that the regulatory role of this additional element is, at least in part, due to some direct interactions with the ligand that extend outside the canonical binding groove and involve residues directly located in this helix^21^.

The role of α3 in the molecular architecture of PDZ3 was also analyzed from a thermodynamic perspective. In fact, it was shown that the deletion of α3 resulted in an increased propensity for this protein to aggregate^19,22^, because of a destabilization of the native state as well as an overall expansion of the polypeptide chain^20^. Here, to obtain a better picture of the role of the C-terminal extension we continue these studies by investigating the folding pathway of the truncated variant of PDZ3 (PDZ3Δα3). A comparative kinetic characterization of the folding of PDZ3 and PDZ3Δα3 reveals that folding is surprisingly robust. The overall kinetic folding mechanism appears similar when the protein is challenged with acidic conditions, different ionic strengths and different temperatures.

## Results and Discussion

In order to test the effect of the α-helical extension in the folding of PDZ3, in analogy to our previous work on this protein family^23–28^, we used a fluorescent pseudo-wild type variant of PDZ3, where a Phe residue is replaced with a Trp in position 337 (F337W). Thus, all experiments on PDZ3 and PDZ3Δα3 were performed using Trp337 as a probe to monitor fluorescence change upon (un)folding.

**Figure 2:**
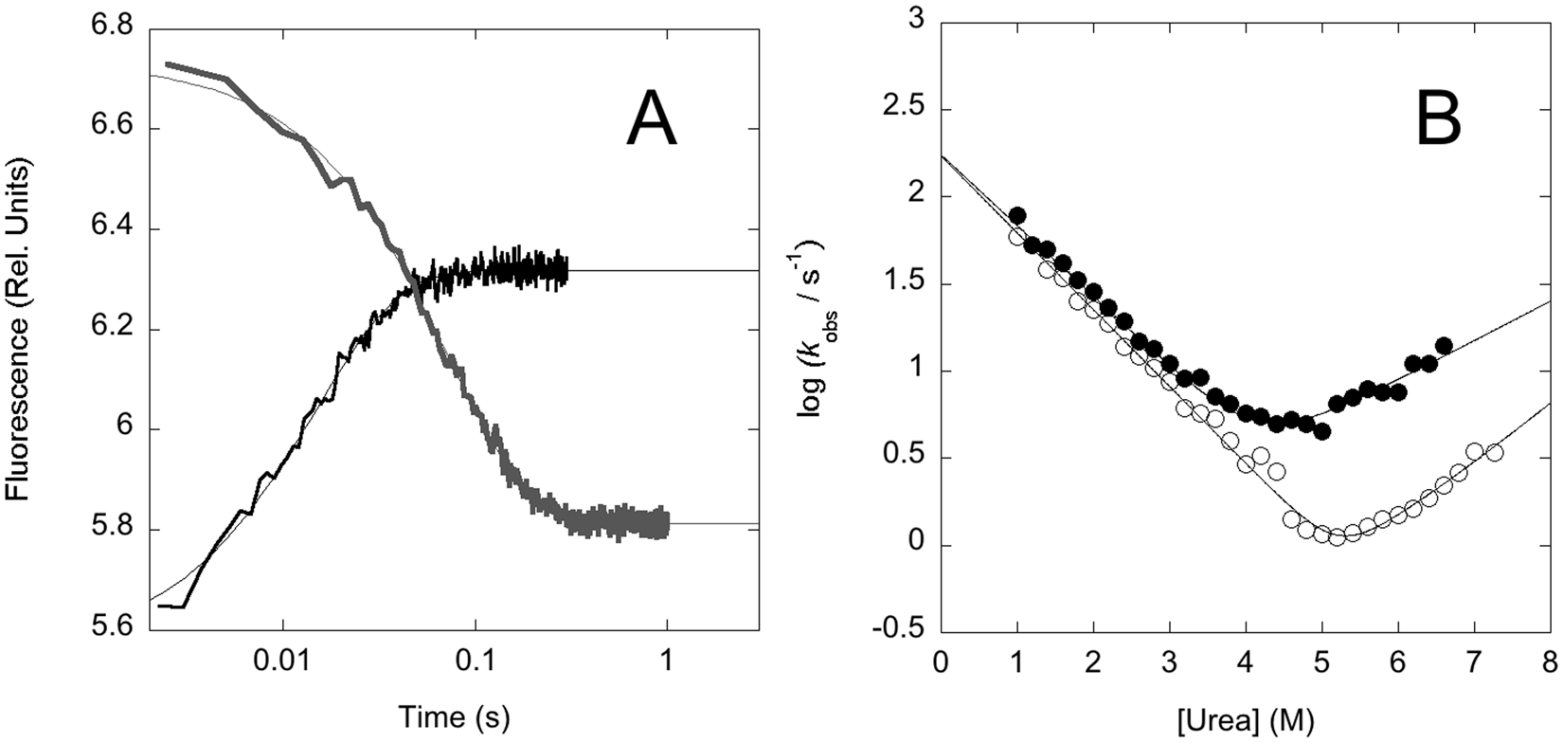
Folding kinetics of PDZ3 and PDZ3Δα3 at neutral pH. Panel A. Representative folding (black) and unfolding (grey) kinetic traces recorded for PDZ3Δα3, the lines are the best fit to a single exponential decay. Panel B. The logarithmic values of the folding and unfolding rate constant of PDZ3 (empty circles) and PDZ3Δα3 (filled circles) were plotted and fitted to a two-state folding equation. The experiments suggest that truncation of α3 decreases the stability of the native state by increasing the unfolding rate constant.

The folding and unfolding of PDZ3 and PDZ3Δα3 were investigated by stopped flow kinetics, by rapidly mixing the native protein with solutions containing different concentrations of urea (unfolding) and by diluting the urea denatured protein with buffer (refolding). Folding was then recorded by monitoring fluorescence emission >320 nm. In all cases, for both PDZ3 and PDZ3Δα3, observed time courses were consistent with a single exponential decay (Fig. 2), suggesting the absence of low-energy transiently populated intermediates^29^.

The semi-logarithmic plot of the observed (un)folding rate constants as a function of urea concentration (chevron plot) for PDZ3 and PDZ3Δα3, recorded in 50 mM sodium phosphate buffer pH 7.2 and at 25 °C, are reported in Fig. 2. It is evident that while the truncation of α3 does not affect the folding arm of the chevron, there is a detectable increase of the unfolding rate constant of about 2.5 fold, corresponding to a relatively marginal destabilization of the native state of about 0.54 kcal mol^−1^. Since the folding rate constant is the same in PDZ3 and PDZ3Δα3, it follows that the deletion of α3 has a negligible effect on the structure of the transition state and that folding of α3 in PDZ3 occurs downhill the main barrier^30^.

Valuable parameters to obtain overall structural features of folding intermediates and transition states are the so-called kinetic *m*-values^31^, which represent the slopes of the folding (*m*_F_) and unfolding (*m*_U_) branches of the chevron plots. The *m* values are correlated with the changes in accessible surface area between the two ground states (native and denatured, respectively) and the transition state between them. The comparison between the chevron plots of PDZ3 and PDZ3Δα3 at neutral pH is reported in Fig. 2 and shows that truncation of the α-helical extension has little effect on the folding and unfolding *m*-values as the apparent branches of the chevron plot appear parallel. Thus, whilst it has been previously observed that PDZ3Δα3 is expanded compared to PDZ3^20^, such expansion cannot be detected from folding kinetics, which appear very similar for the two proteins.

**Figure 3:**
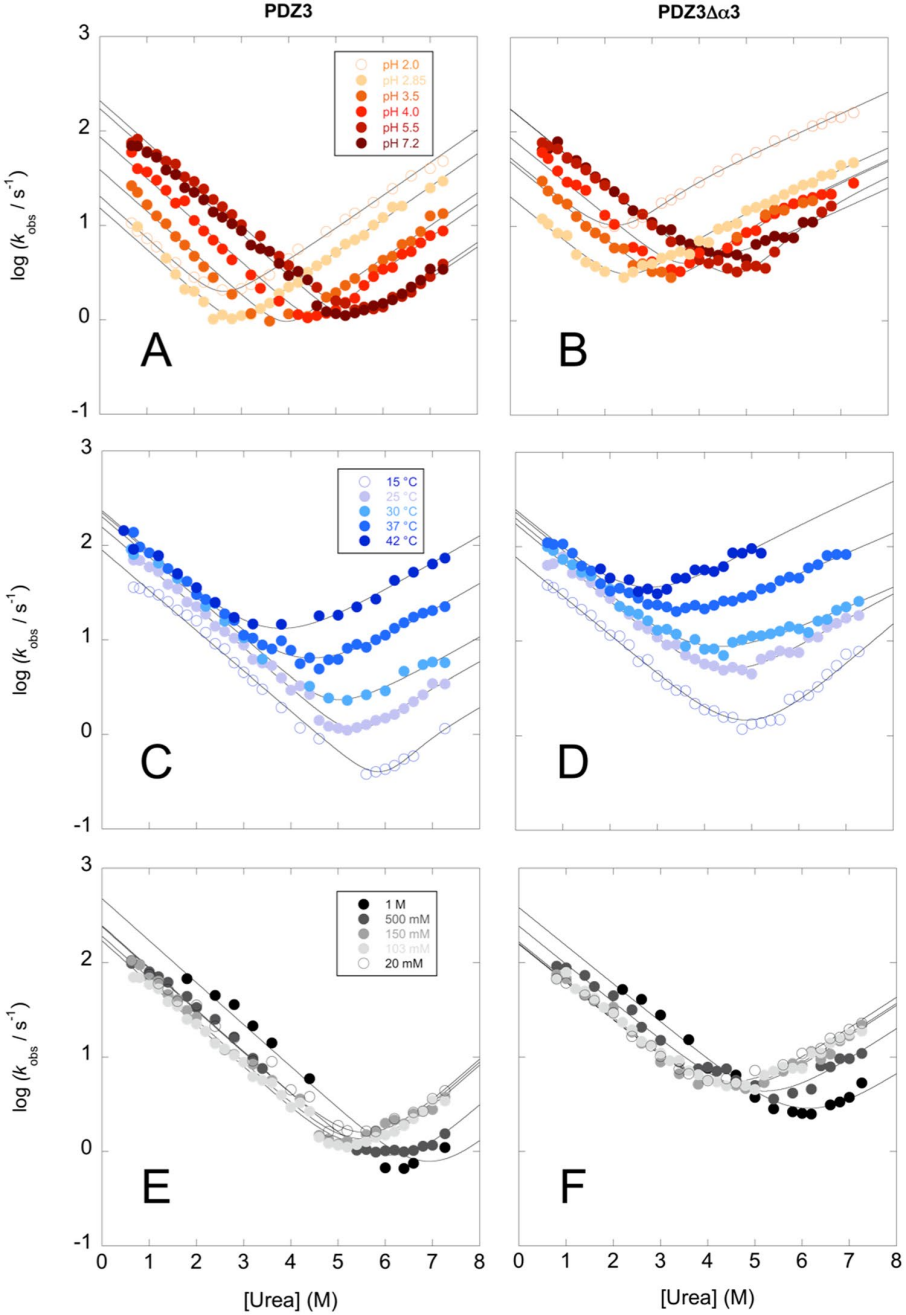
Folding kinetics of PDZ3 (left) and PDZ3Δα3 (right) at different experimental conditions. Panel A and B: Chevron plots of the logarithm of unfolding and folding rate constants measured at different pH ranging from 2.0 to 7.2 at 25 °C. The lines are the best fit to a three-state model (Equation 1) with shared folding and unfolding *m*-values. Panel C and D: Chevron plots of the logarithm of unfolding and folding rate constants measured at different temperatures ranging from 15 to 42 °C and in 50 mM sodium-phosphate buffer at pH 7.2. Panel E and F: Chevron plots of the logarithm of the unfolding and folding rate constants measured in sodium-phosphate buffer at pH 7.2 with different ionic strengths ranging from 20 mM to 1 M and at 25 °C. The lines in panel C, D, E and F are the best fit to a two-state chevron.

To obtain a comprehensive comparison of the folding mechanisms of the two proteins, we investigated the folding at different pH conditions (ranging from pH 2.0 to 7.2), ionic strengths (from 20 mM to 1 M) and temperatures (from 15 °C to 42 °C). The resulting experiments are shown in Fig. 3. In a previously published series of papers^7,25,26,28,32,33^, we have shown how the folding of PDZ domains may vary from an apparent two-state (V-shaped chevron plot) to more complex scenarios (curved chevron plot), under different experimental conditions (e.g. different pH values or in the presence of a stabilizing salt) or with site-directed conservative mutants. To rationalize these apparently different mechanisms, we showed that a global quantitative analysis of all folding data can be described in terms of a reaction scheme involving a single set of intermediates and transition states. In this context, the presence/absence of curvatures in the chevron plot would arise from selective (de)stabilization of the activation barriers and ground states, when varying solvent composition or protein sequence.

In the case of PDZ3, we observed that, whilst the chevron plot is essentially V-shaped at physiological conditions, it displays a detectable curvature when the protein is destabilized by lowering pH or by site-directed mutagenesis^32^. Analogously, in this work, we observed a clear curvature in the unfolding arms of the chevron plots of PDZ3Δα3, whilst in the case of PDZ3 a deviation from linearity could be clearly observed at acidic pH conditions. This finding suggests the presence of a high-energy folding intermediate between two different transition states, namely a denatured-like transition state *TS1* and a native like transition state *TS2*. At increasing denaturant concentrations there is a switch in rate limiting transition state from *TS1* to *TS2*. The folding of both PDZ3 and PDZ3Δα3 carries the signature of such an intermediate, indicating that the truncation of the C-terminal extension does not contribute to a detectable distortion of the overall folding mechanism.

By applying a three-state model^34^ the data reported in Fig. 3 were used to calculate the folding rate constant *k*_F_ and two different unfolding rate constants *k*_U1_ and *k*_U2_ associated with the two transition states *TS1* and *TS2*, respectively. Furthermore, quantitative analysis of the chevron plots allows measuring the associated Tanford β-values, which reflect the relative positions of *TS1* and *TS2* along the reaction coordinate with regard to compactness. Tanford β-values for PDZ3Δα3 were 0.4 ± 0.1 for the denatured like transition state *TS1* and 0.82 ± 0.03 for the native-like *TS2*. These values appear very similar to those of PDZ3 (0.5 ± 0.2 and 0.85 ± 0.07), showing that truncation of α3 does not distort the overall folding transition state structures. In experiments performed as a function of temperature and ionic strength, we could not observe a detectable curvature in the unfolding arm of the chevron plot for either PDZ3 or PDZ3Δα3. This observation indicates that, in both proteins, the transition state *TS1* represents the rate limiting step at every concentration of urea experimentally accessible and no change in rate determining barrier can be detected. Accordingly, those data were fitted to a standard two-state chevron^29^.

**Figure 4:**
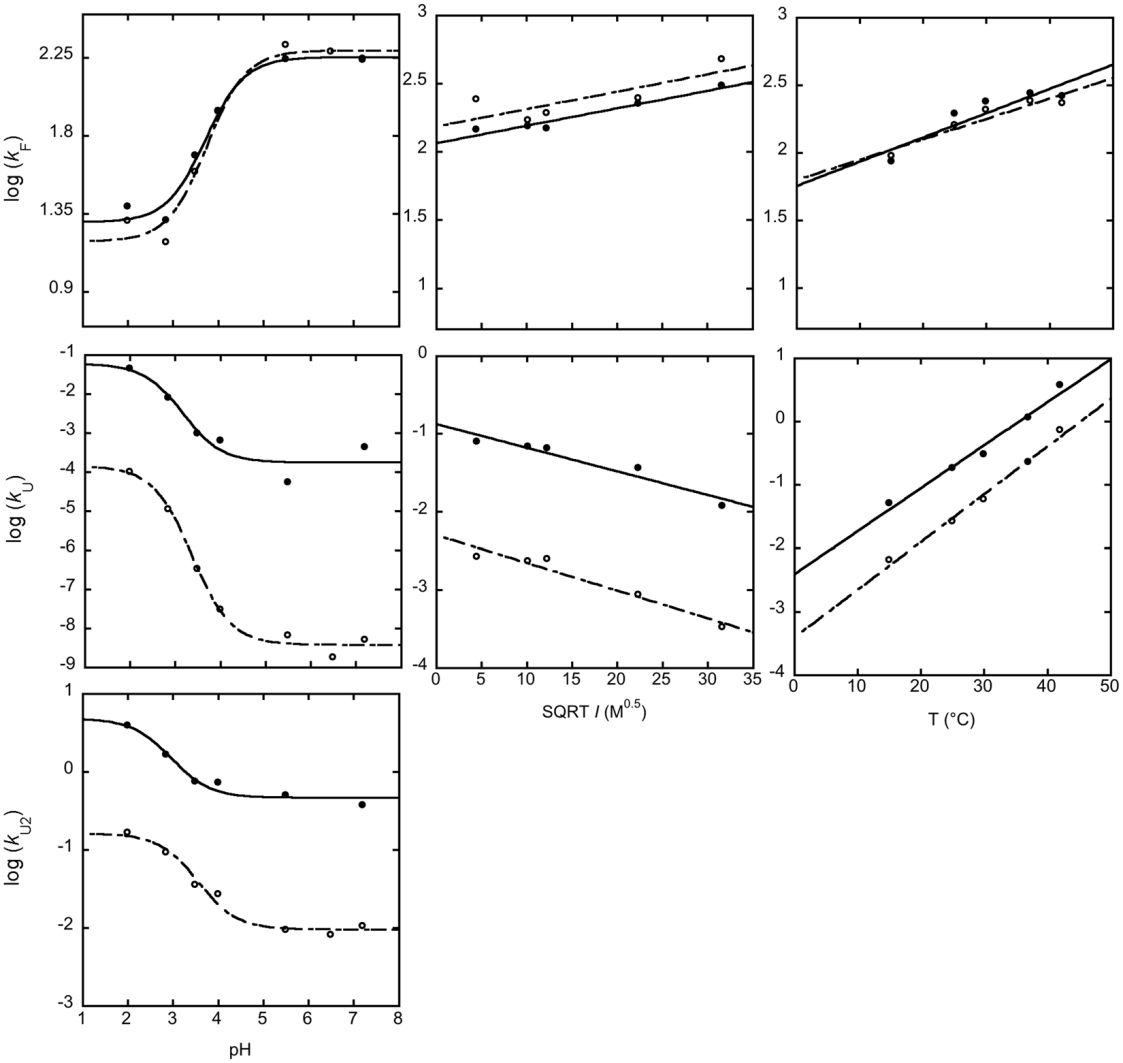
Comparison of folding and unfolding rate constants calculated in the absence of denaturant of PDZ3 (empty circles) and PDZ3Δα3 (filled circles) at different experimental conditions. The rate constants were obtained by analysis of the chevron plots reported in Fig. 3. The folding rate constant *k*_F_ is very similar for both PDZ3 and PDZ3Δα3 at all the experimental conditions explored. Data recorded at different pH conditions, left column, where fitted to the Henderson–Hasselbalch equation, returning a single transition with a robust apparent p*K*_a_ of about 3.5. In all cases, an increase in the microscopic unfolding rate constant could be observed, consistent with a destabilization of the native state. The overall dependence of the unfolding rate constants were however unaffected by the truncation of α3.

**Figure 5:**
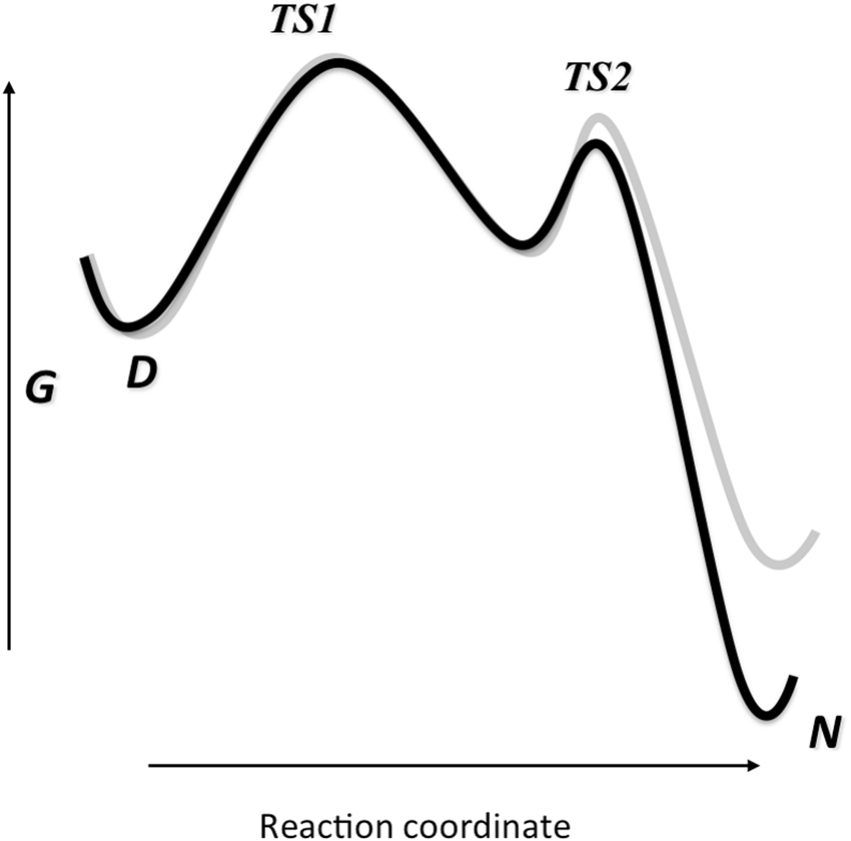
Energy diagram for the folding of of PDZ3 (black) and PDZ3Δα3 (grey). Deletion of the of helix α3 affects slightly the stability of the native state without altering substantially the major folding pathway, which appears rather robust to the truncation.

The dependence of the microscopic folding and unfolding rate constants on ionic strength, temperature and pH are reported in Fig. 4. In all cases, the folding rate constant *k*_F_ is the same within experimental error for both of PDZ3 and PDZ3Δα3 when varying the experimental conditions, indicating that the structure of the folding transition state is very robust. Furthermore, the overall behaviour of both unfolding rate constant *k*_U1_ and *k*_U2_ appear very similar, displaying the same dependence on temperature, ionic strength and pH. On the basis of these observations we conclude that the overall folding mechanism of PDZ3 is not substantially perturbed and, while destabilized, is not affected by the deletion of helix α3. A schematic energy diagram summarizing the effect of the deletion of helix α3 is presented in Fig. 5.

## Conclusions

It has been previously suggested that the presence of an additional helix, the C-terminal α3 extension in PDZ3 is critical in modulating its function as well as aggregation properties^14,17,19–22^. Furthermore, truncation of α3 has been shown to lead to an overall expansion of the polypeptide chain, while exhibiting no significant changes in the overall structure^20^. In this work, we completed these analyses by comparing the folding and unfolding of PDZ3 and PDZ3Δα3 under a variety of experimental conditions. Our data reveal that the mechanism by which the denatured state of PDZ3 achieves its native conformation is rather robust. In fact, whilst a destabilization of the native state could be observed, there is no effect in the folding rate constant at any of the experimental conditions explored, indicating that the C-terminal extension α3 forms downhill of the main barrier for folding. This finding appears to contrast, at least in part, the observed stabilization of an equilibrium intermediate in PDZ3Δα3, which leads to a more complex folding behaviour as well as to an increased propensity for the protein to aggregate^19,35^. It is important to notice, however, that the intermediate previously identified by Murciano-Calles *et al*. by differential scanning calorimetry, is most likely a distinct species from the meta-stable state responsible for the curvature of the chevron plot of PDZ3, which is a high-energy species that never accumulates. On the basis of our experimental work, we conclude that the main folding transition state is by and large unaffected by the deletion of α3 and the overall folding mechanism of PDZ3 appears robust. Future work based on protein engineering will further elucidate the mechanistic details of the role of helix α3 in the folding and function of PDZ3.

## Methods

### Protein Expression and Purification

All experiments were performed with a pseudo-wild type (pWT) PSD-95 PDZ3 containing a Trp at position 337. The constructs pWT-6His-PDZ3F337W and pWT-6His-PDZ3F337W-Δα3 coding for residues 309 to 402 and 309 to 394, respectively, of PSD-95 PDZ3 were used for protein expression and purification as previously described^27^. All experiments were performed on pure protein samples as judged by SDS-PAGE and MALDI mass spectrometry.

### Kinetics Experiment*s*

Rapid mixing for kinetics folding and unfolding experiments were carried out on a stopped-flow device (Pi-star, Applied Photophysics, Leatherhead, UK) with an excitation wavelength of 280 nm and a 320 nm cut-off glass filter to measure fluorescence emission. All kinetic folding experiments were performed with 2 μM final concentration of protein and urea concentrations ranging from 0.4 to 7.27 M. Temperature dependence studies were made at 15, 25, 30, 37 and 42 °C in 50 mM sodium-phosphate pH 7.2. For the pH and ionic strength dependence studies the temperature was set at 25 °C. Buffers used for pH dependence studies were: 50 mM sodium phosphate at pH 2.0, 6.5, and 7.5; 50 mM sodium formate at pH 2.85, 3.5, and 4.0; and 50 mM sodium acetate at pH 5.5. Ionic strength dependence experiments were made with varying concentrations of sodium phosphate buffer at pH 7.2 and addition of sodium chloride to obtain ionic strengths ranging from 20 mM to 1 M.

### Data Analysis

Kinetic traces were fitted with a single exponential decay using Applied Photophysics software to obtain the observed rate constant *k*_obs_. The logarithmic values of *k*_obs_ were plotted versus urea concentration (chevron plot) and a equation describing a three-state folding reaction was used to estimate microscopic folding rate constants:

1 $${k}_{obs}={k}_{F}+\frac{{k}_{U1}}{1+{K}_{part}}$$

where *k*_F_ is the folding rate constant, *K*_part_ is a partition constant between *k*_U1_ and *k*_U2_, the two unfolding rate constants referring to the denatured-like and native-like transition states *TS1* and *TS2*, respectively. *k*_U2_, is equal to *k*_U1_/*K*_part_. The dependence of the logarithm of *k*_F_, *k*_U1_ and *K*_part_ was assumed to be linearly dependent on denaturant concentration, with a respective slope denoted as *m*_F_, *m*_U1_ and *m*_part_, respectively. The Tanford β-values were calculated as β_Ti_ = *m*_i_/*m*_D-N_ where *m*_i_ is the *m* value at a given state *i*, and *m*_D-N_ the total *m* value between the denatured and native state. Whilst the experiments performed as a function of pH allowed to infer the presence of both transition states for both PDZ3 and PDZ3Δα3, for data recorded as a function of temperature and increasing ionic strength we could not identify a major curvature in the chevron plots. Accordingly, in those cases, data were fitted to a standard two-state chevron^29^.

## References

[1] Kim E, Sheng M (2004). PDZ domain proteins of synapses. Nat. Rev. Nuerosci..

[2] Luck K, Charbonnier S, Trave G (2012). The emerging contribution of sequence context to the specificity of protein interactions mediated by PDZ domains. FEBS Letters.

[3] van Ham M, Hendriks W (2003). PDZ domains-glue and guide. Mol. Biol. Rep..

[4] Ye F, Zhang M (2013). Structures and target recognition modes of PDZ domains: recurring themes and emerging pictures. Biochem. J..

[5] Ivarsson Y (2012). Plasticity of PDZ domains in ligand recognition and signaling. FEBS Lett..

[6] Chi CN, Bach A, Strømgaard K, Gianni S, Jemth P (2012). Ligand binding by PDZ domains. Biofactors.

[7] Jemth P, Gianni S (2007). PDZ domains: folding and binding. Biochemistry.

[8] Doyle DA (1996). Crystal structures of a complexed and peptide-free membrane protein-binding domain: molecular basis of peptide recognition by PDZ. Cell.

[9] Feng W, Zhang M (2009). Organization and dynamics of PDZ-domain-related supramodules in the postsynaptic density. Nat. Rev. Nuerosci..

[10] Wang CK, Pan L, Chen J, Zhang M (2010). Extensions of PDZ domains as important structural and functional elements. Protein Cell..

[11] Gerek ZN, Ozkan SB (2011). Change in allosteric network affects binding affinities of PDZ domains: analysis through perturbation response scanning. Plos Comput. Biol..

[12] Morra G, Genoni A, Colombo G (2014). Mechanisms of Differential Allosteric Modulation in Homologous Proteins: Insights from the Analysis of Internal Dynamics and Energetics of PDZ Domains. J. Chem. Theory Comput..

[13] Hultqvist G (2013). Energetic pathway sampling in a protein interaction domain. Structure.

[14] Fuentes EJ, Der CJ, Lee AL (2004). Ligand-dependent dynamics and intramolecular signaling in a PDZ domain. J. Mol. Biol..

[15] Lockless SW, Ranganathan R (1999). Evolutionarily conserved pathways of energetic connectivity in protein families. Science.

[16] Kong Y, Karplus M (2009). Signaling pathways of PDZ2 domain: a molecular dynamics interaction correlation analysis. Proteins.

[17] Petit CM (2009). Hidden dynamic allostery in a PDZ domain. Proc. Natl. Acad. Sci. USA.

[18] Gianni S (2011). Sequence specific long-range networks in PDZ domains tune their binding selectivity. J. Biol. Chem..

[19] Murciano-Calles J, Marin-Argany M, Cobos ES, Villegas S, Martinez JC (2014). The impact of extra-domain structures and post-translational modifications in the folding/misfolding behaviour of the third PDZ domain of MAGUK neuronal protein PSD-95. Plos One.

[20] Law AB, Sapienza PJ, Zhang J, Zuo X, Petit CM (2017). Native State Volume Fluctuations in Proteins as a Mechanism for Dynamic Allostery. J. Am. Chem. Soc..

[21] Chi CN (2012). Interactions outside the boundaries of the canonical binding groove of a PDZ domain influence ligand binding. Biochemistry.

[22] Murciano-Calles J, Güell-Bosch J, Villegas S, Martinez JC (2016). Common features in the unfolding and misfolding of PDZ domains and beyond: the modulatory effect of domain swapping and extra-elements. Sci. Rep..

[23] Chi CN (2009). A sequential binding mechanism in a PDZ domain. Biochemistry.

[24] Chi CN, Engstrom A, Gianni S, Larsson M, Jemth P (2006). Two conserved residues govern the salt and pH dependencies of the binding reac- tion of a PDZ domain. J. Biol. Chem..

[25] Chi CN (2007). A conserved folding mechanism for PDZ domains. FEBS Lett..

[26] Gianni S (2005). Kinetic folding mechanism of PDZ2 from PTP-BL. Prot. Eng. Des. Sel..

[27] Gianni S (2005). & P., J. The kinetics of PDZ domain-ligand interactions and implications for the binding mechanism. J. BIol. Chem..

[28] Ivarsson Y (2007). An On-pathway Intermediate in the Folding of a PDZ Domain. J. Biol. Chem..

[29] Jackson SE, Fersht AR (1991). Folding of chymotrypsin inhibitor 2. 1. Evidence for a two-state transition. Biochemistry.

[30] Fersht, A. Structure and Mechanism in Protein Science. *Freeman W.H. and Co New York*, (1999).

[31] Myers JK, Pace CN, Scholtz JM (1995). Denaturant m values and heat capacity changes: relation to changes in accessible surface areas of protein unfolding. Protein Sci..

[32] Calosci N (2008). Comparison of successive transition states for folding reveals alternative early folding pathways of two homologous proteins. Proc. Natl. Acad. Sci. U S A.

[33] Hultqvist G, Pedersen SW, Chi CN, Strømgaard K, Gianni S (2012). & P., J. An expanded view of the protein folding landscape of PDZ domains. Biochem. Biophys. Res. Commum..

[34] Parker MJ, Spencer J, Clarke AR (1995). An integrated kinetic analysis of intermediates and transition states in protein folding reactions. J Mol Biol.

[35] Murciano-Calles J, Martinez JC, Marin-Argany M, Villegas S, Cobos ES (2014). A thermodynamic study of the third PDZ domain of MAGUK neuronal protein PSD-95 reveals a complex three-state folding behavior. Biophys. Chem..

